# Tobacco smoking and mortality among Aboriginal and Torres Strait Islander adults in Australia

**DOI:** 10.1093/ije/dyaa274

**Published:** 2021-01-25

**Authors:** Katherine A Thurber, Emily Banks, Grace Joshy, Kay Soga, Alexandra Marmor, Glen Benton, Sarah L White, Sandra Eades, Raglan Maddox, Tom Calma, Raymond Lovett

**Affiliations:** 1 National Centre for Epidemiology and Population Health, Research School of Population Health, Australian National University, Acton, ACT, Australia; 2 Sax Institute, Ultimo, NSW, Australia; 3 Quit Victoria, Melbourne, VIC, Australia; 4 Curtin Medical School, Bentley, WA, Australia; 5 University of Canberra, Bruce, ACT, Australia

**Keywords:** Tobacco, smokers, mortality, premature, smoking cessation, adult, Australia

## Abstract

**Background:**

Despite generally high smoking prevalences, stemming from colonization, the relationship of smoking to mortality has not been quantified reliably in an Indigenous population. We investigate smoking and mortality among Aboriginal and Torres Strait Islander adults in Australia, where current adult daily smoking prevalence is 40.2%.

**Methods:**

A prospective study of 1388 cardiovascular disease- and cancer-free Aboriginal adults aged ≥45 years, of the 267 153 45 and Up Study participants randomly sampled from the New South Wales general population over 2006–09. Questionnaire and mortality data were linked (through the Centre for Health Record Linkage) to mid-2019. Adjusted hazard ratios (called relative risks, RRs) for all-cause mortality—among current- and past- versus never-smokers—were estimated overall, by smoking intensity and by age at cessation. Smoking-attributable fractions and associated deaths were estimated.

**Results:**

Over 14 586 person-years’ follow-up (median 10.6 years), 162 deaths accrued. Mortality RRs [95% confidence interval (CI)] were 3.90 (2.52–6.04) for current- and 1.95 (1.32–2.90) for past- versus never-smokers, with age heterogeneity. RRs increased with smoking intensity, to 4.29 (2.15–8.57) in current-smokers of ≥25 cigarettes/day. Compared with never-smokers, RRs were 1.48 (0.85–2.57) for those quitting at <45 years of age and 2.21 (1.29–3.80) at 45–54 years. Never-smokers lived an average >10 years longer than current-smokers. Around half of deaths among adults aged ≥45 years were attributable to smoking, exceeding 10 000 deaths in the past decade.

**Conclusions:**

In this population, >80% of never-smokers would survive to 75 years, versus ∼40% of current-smokers. Quitting at all ages examined had substantial benefits versus continuing smoking; those quitting before age 45 years had mortality risks similar to never-smokers. Smoking causes half of deaths in older Aboriginal and Torres Strait Islander adults; Indigenous tobacco control must receive increased priority.

##  

**Key Messages**

- Our study provides the first direct estimates of smoking-attributable mortality for Aboriginal and Torres Strait Islander peoples, and the first estimates for any Indigenous population internationally that account for pre-existing disease.
- Smoking causes half of deaths in older Aboriginal and Torres Strait Islander adults, and over one-third of all deaths in the population.
- Smoking’s effect has been underestimated, in large part due to the lack of relevant data and analyses; high-quality data are needed for other Indigenous populations.
- This study is a world first, and shows a clear way forward for major improvement in Indigenous health: reducing tobacco use would have a tremendous impact at the population level.
- Sustained, comprehensive, and population-wide tobacco control for Aboriginal and Torres Strait Islander peoples is required to reverse potential tobacco-related harms, and then end this epidemic.

## Introduction

The lasting impacts of colonial processes, as well as tobacco marketing, have entrenched commercial tobacco use in the Australian Aboriginal and Torres Strait Islander population, and in many Indigenous populations globally.^1–5^ Although substantially decreased over the past 15 years, Aboriginal and Torres Strait Islander adult daily smoking prevalence remains high at 40.2%,^6^ with smoking the single leading contributor to burden of disease.^7^ There is contemporary, direct evidence on smoking-attributable mortality (SAM) for the total Australian population,^8^ but not for the Aboriginal and Torres Strait Islander population.^1^ Accurate quantification of SAM in this population is required to quantify and communicate smoking’s risks, and to inform policy and practice decision making.

Smoking causes premature death. The magnitude of the relationship between smoking and mortality varies between population groups and over time, according to factors including smoking prevalence, duration and intensity.^9^ The Aboriginal and Torres Strait Islander smoking epidemic has unique features, including protracted duration and high prevalence among females and males.^1^ Further, the background mortality rate is substantially higher than in the total Australian population.

Thus far, quantifications of SAM in the Aboriginal and Torres Strait Islander population have used indirect methods, incorporating relative risk estimates from other populations.^1^ However, population-specific estimates are needed. We quantify the relationship of smoking to mortality in a cohort of Aboriginal and Torres Strait Islander adults, and quantify SAM in the national population, overall and by age group and sex.

## Methods

Ethics approval for the 45 and Up Study was provided by the University of New South Wales Human Research Ethics Committee (HREC). Ethics approval for this analysis was provided by the NSW Population and Health Services Research Ethics Committee (12/CIPHS/31), the Aboriginal Health and Medical Research Council (1006/14) and the Australian National University HREC (2012/504).

### Study population

The Sax Institute’s 45 and Up Study is a cohort study of 267 153 men and women aged ≥45 years, randomly sampled from the general population of New South Wales (NSW), Australia, using the Department of Human Services (previously Medicare Australia) enrolment database. Regional and remote areas and those aged ≥80 were over-sampled. Individuals joined the study by completing postal questionnaires over 2006–09 and consenting to follow-up through repeated surveys and data linkage.^10^

We analysed data from participants completing the baseline questionnaire who self-identified as Aboriginal and/or Torres Strait Islander, with valid data on age, recruitment date and smoking status, who were aged ≥45 at baseline, and whose data could be successfully linked (Supplementary Figure S1, available as Supplementary data at *IJE* online). Hereafter cohort participants are respectfully referred to as Aboriginal, as Aboriginal peoples comprise the vast majority of the NSW Indigenous population and are the original inhabitants of the area.

To minimize the potential impact of changes in smoking behaviour and higher mortality among those with baseline illness (‘sick quitter’ effect), we excluded participants self-reporting previous doctor-diagnosed cardiovascular disease (heart disease, stroke or blood clot) or cancer (excluding melanoma and/or non-melanoma skin cancer).

### Data

Remoteness and area-level disadvantage were from participants’ postcodes. All other variables were from self-reported baseline questionnaire responses (Supplementary Table S1, available as Supplementary data at *IJE* online). Baseline questionnaire data were probabilistically linked to data on fact of death from the NSW Registry of Births, Deaths and Marriages (1 January 2006–31 March 2019), and cause of death from the Cause of Death Unit Record File held by the NSW Ministry of Health Secure Analytics for Population Health Research and Intelligence and National Death Index (1 January 2006–30 November 2017).

### Statistical methods

Mortality rates since baseline were calculated for past-smokers and current-smokers compared with never-smokers. Hazard ratios (hereafter referred to as relative risks, RRs) and 95% confidence intervals (CIs) for mortality were estimated using Cox regression modelling, with age as the underlying time variable.

The RR of dying during follow-up, compared with never-smokers, was quantified by smoking intensity among current-smokers, and by age at cessation among past-smokers—restricted to those quitting by age 55, to reduce potential bias due to the ‘sick quitter’ effect. Results are adjusted for age group and sex (RR^1^), and additionally adjusted for remoteness and education (RR^2^, reported in text) and presented stratified by sex and geographical remoteness.

Sensitivity analyses were conducted: using follow-up duration as the underlying time scale; additionally adjusting for alcohol intake; reclassifying past-smokers as current-smokers if they had quit <3 years before baseline (capturing those who quit due to becoming sick); and with the reference category as current-smokers or the highest smoking intensity category, as relevant. Proportionality assumptions were verified using Schoenfeld residuals. All statistical tests were two-sided, with alpha = 0.05. Hypothetical survival curves for current-smokers and never-smokers were plotted for illustrative purposes, demonstrating the absolute effects of observed RRs.

Cause of death was categorized into broad groupings according to the International Statistical Classification of Diseases and Related Health Problems Version 10, Australian Modification (ICD-10-AM) (Supplementary Table S2, available as Supplementary data at *IJE* online), and compared across smoking categories, to examine the extent to which deaths were due to conditions made appreciably more probable by smoking.^11^

Smoking-attributable fractions (SAF) for all-cause mortality overall and by age group and sex were calculated by applying sex-combined, age group-specific RRs based on global evidence of age (but not sex) differences in the smoking-mortality relationship.^8^^,^^12^ We calculated the SAF for all-cause mortality during cohort follow-up (2009–18), assuming 90% of excess deaths were attributable to smoking,^11^ with 80% the lower and 100% the upper bound. As smoking prevalence ≥45 years did not change materially between 1998 and 2008,^13^ national smoking prevalence estimates from 2008^14^ were used to allow an aetiologically appropriate lag time between exposure and outcome.^15^ Near-future SAF was re-calculated using 2018/19 prevalence.^6^

We used the SAF to calculate the absolute number of smoking-attributable deaths of Aboriginal and Torres Strait Islander people aged ≥45 years over the past decade. Deaths in each age-sex category were extracted from the Australian Bureau of Statistics’ ABS.Stat.^16^ We estimated the proportion of all deaths (all ages) caused by smoking, under the conservative assumption that smoking caused no deaths under 45 years, and under the assumption that 35–44 year olds experienced the same mortality RR as 45–54 year olds—an equivalent level of risk cannot be excluded based on international evidence.^17^ Analyses were conducted using SAS^®^ version 9·4 and Excel.

### Engagement

Consistent with ethical principles, the development, analysis, interpretation and dissemination of study findings included active and meaningful Indigenous engagement. The study includes Indigenous authorship and leadership. Findings, interpretations and messaging were discussed with the Thiitu Tharrmay Research Reference Group, which informed our strengths-based messaging and engagement strategy. Organizations involved in policy and in providing health care and tobacco control have been engaged to discuss and disseminate findings.

## Results

The final sample included 1388 Aboriginal adults (Supplementary Figure S1, available as Supplementary data at *IJE* online), 41.9% (*n* = 582) of whom were never-smokers, 36.6% (*n* = 508) past-smokers and 21.5% (*n* = 298) current-smokers at baseline (Table 1).

**Table 1:** Sociodemographic characteristics of Aboriginal participants in the 45 and Up Study, overall and by smoking status

	Smoking status						Total	
	Current		Past		Never			
	%	(*n*)	%	(*n*)	%	(*n*)	%	(*n*)
Overall	21.5	(298)	36.6	(508)	41.9	(582)	100	(1388)
Age (years)								
45–64	91.9	(274)	80.9	(411)	81.8	(476)	83.6	(1161)
65–74	6.4	(19)	14.6	(74)	14.3	(83)	12.7	(176)
≥75	1.7	(5)	4.5	(23)	4.0	(23)	3.7	(51)
Gender								
Male	45.3	(135)	49.6	(252)	37.1	(216)	43.4	(603)
Female	54.7	(163)	50.4	(256)	62.9	(366)	56.6	(785)
Remoteness								
Major Cities	35.6	(106)	38.2	(194)	38.0	(221)	37.5	(521)
Inner regional	36.6	(109)	41.1	(209)	38.3	(223)	39.0	(541)
Outer regional/remote	27.2	(81)	18.9	(96)	21.6	(126)	21.8	(303)
Missing	0.7	(2)	1.8	(9)	2.1	(12)	1.7	(23)
Education								
No school certificate	34.6	(103)	27.4	(139)	23.7	(138)	27.4	(380)
School or other certificate/diploma	54.4	(162)	54.7	(278)	55.8	(325)	55.1	(765)
University degree or higher	8.1	(24)	14.4	(73)	17.0	(99)	14.1	(196)
Missing	3.0	(9)	3.5	(18)	3.4	(20)	3.4	(47)
Annual household income (ASD)								
<$20 000	36.2	(108)	28.5	(145)	21.8	(127)	27.4	(380)
$20 000-$39 999	17.4	(52)	15.7	(80)	17.0	(99)	16.6	(231)
$40 000-$69 999	11.1	(33)	16.5	(84)	18.4	(107)	16.1	(224)
≥$70 000	11.1	(33)	16.9	(86)	19.6	(114)	16.8	(233)
Missing	24.2	(72)	22.2	(113)	23.2	(135)	23.1	(320)
Private health insurance								
No private health insurance	83.2	(248)	65.7	(334)	54.3	(316)	64.7	(898)
Hospital/DVA insurance	16.8	(50)	34.3	(174)	45.7	(266)	35.3	(490)
Alcoholic drinks per week								
None	43.0	(128)	39.0	(198)	44.2	(257)	42.0	(583)
1–14	31.9	(95)	38.4	(195)	40.7	(237)	38.0	(527)
15 or more	20.1	(60)	19.5	(99)	8.2	(48)	14.9	(207)
Missing	5.0	(15)	3.1	(16)	6.9	(40)	5.1	(71)
Physical activity tertile								
First tertile (low activity)	28.5	(85)	27.2	(138)	27.1	(158)	27.4	(381)
Second tertile	32.2	(96)	33.3	(169)	37.6	(219)	34.9	(484)
Third tertile (high activity)	34.2	(102)	33.7	(171)	28.5	(166)	31.6	(439)
Missing	5.0	(15)	5.9	(30)	6.7	(39)	6.1	(84)
Body mass index								
15–19.9	8.7	(26)	1.6	(8)	1.7	(10)	3.2	(44)
20–24.9	26.2	(78)	18.7	(95)	18.4	(107)	20.2	(280)
25–29.9	28.5	(85)	32.1	(163)	35.1	(204)	32.6	(452)
30–50	23.5	(70)	37.2	(189)	32.8	(191)	32.4	(450)
Missing	13.1	(39)	10.4	(53)	12.0	(70)	11.7	(162)
Functional limitation								
No limitation	53.7	(160)	56.1	(285)	63.2	(368)	58.6	(813)
Limitation	26.8	(80)	24.4	(124)	18.4	(107)	22.4	(311)
Missing	19.5	(58)	19.5	(99)	18.4	(107)	19.0	(264)

DVA, Department of Veterans’ Affairs.

Median age at baseline was 54.5 years, with the majority of participants aged 45–64 years (83.6%); 56.6% were female. It was more common for never-smokers than current-smokers to have high education, high income, private health insurance and high body mass index, and less common for never-smokers to be heavy drinkers or have physical functional limitation. Around half of all past-smokers quit at age ≤44 (Table 2).

**Table 2:** Smoking characteristics of current and past-smokers, overall and by sex

	Median within category	Total		Sex			
				Males		Females	
		%	(*n*)	%	(*n*)	%	(*n*)
Current-smokers							
Age at smoking initiation (years)							
<16	14 years	44.6	(133)	51.9	(70)	38.7	(63)
16-20	18 years	36.2	(108)	32.6	(44)	39.3	(64)
≥21	25 years	12.1	(36)	8.1	(11)	15.3	(25)
Missing	—	7.0	(21)	7.4	(10)	6.7	(11)
Smoking duration (years)							
<25	19 years	≤ 5.6	(≤ 17)	≤ 3.6	(≤ 5)	≤ 7.3	(≤ 12)
25–39	34 years	63.8	(190)	57.8	(78)	68.7	(112)
≥40	45 years	24.2	(72)	31.9	(43)	17.8	(29)
Missing	—	≤ 7.6	(≤23)	≤ 8.0	(≤11)	≤ 7.3	(≤ 12)
Smoking intensity (cigarettes/day)							
1–14	10 cigarettes/day	26.5	(79)	22.2	(30)	30.1	(49)
15–24	20 cigarettes/day	46.0	(137)	43.0	(58)	48.5	(79)
≥25	30 cigarettes/day	24.2	(72)	30.4	(41)	19.0	(31)
Missing	—	3.4	(10)	4.4	(6)	2.5	(4)
Past-smokers							
Age at smoking initiation (years)							
<16	14 years	33.5	(170)	36.1	(91)	30.9	(79)
16-20	17 years	45.5	(231)	45.6	(115)	45.3	(116)
≥21	24 years	13.4	(68)	11.9	(30)	14.8	(38)
Missing	—	7.7	(39)	6.3	(16)	9.0	(23)
Age at smoking cessation (years)							
<30	24 years	19.3	(98)	17.9	(45)	20.7	(53)
30–44	38 years	33.5	(170)	34.5	(87)	32.4	(83)
45–54	49 years	26.6	(135)	27.0	(68)	26.2	(67)
≥55	60 years	11.8	(60)	13.5	(34)	10.2	(26)
Missing	—	8.8	(45)	7.1	(18)	10.5	(27)
Smoking duration (years)							
<25	13 years	44.7	(227)	46.8	(118)	42.6	(109)
25–39	30 years	35.2	(179)	33.3	(84)	37.1	(95)
≥40	45 years	9.1	(46)	11.5	(29)	6.6	(17)
Missing	—	11.0	(56)	8.3	(21)	13.7	(35)
Smoking intensity (cigarettes/day)							
1–14	10 cigarettes/day	33.7	(171)	25.8	(65)	41.4	(106)
15–24	20 cigarettes/day	34.8	(177)	38.1	(96)	31.6	(81)
≥25	30 cigarettes/day	30.1	(153)	34.1	(86)	26.2	(67)
Missing	—	1.4	(7)	2.0	(5)	0.8	(2)

Over 14 586 person-years of follow-up (median 10.6 years), 162 deaths accrued, giving a crude mortality rate of 11.11 per 1000 person-years (Table 3). Overall, compared with never-smokers, RRs of dying during follow-up were 1.95 (95% CI : 1.32–2.90) in past-smokers and 3.90 (2.52–6.04) in current-smokers, with significant heterogeneity by age: RRs for current- versus never-smokers were 6.92 (2.98–16.04) for 45–64, 5.51 (2.36–13.15) for 65–74 and 1.97 (0.83–4.66) for ≥75 years.

**Table 3:** Absolute rates and relative risks of all-cause mortality among Aboriginal current- and past-smokers in the 45 and Up Study, relative to never-smokers, overall and by age group

		Deaths	*P*-years	Crude rate	RR^1^ (95% CI)	RR^2^ (95% CI)
Total		162	14 586	11.11	—	—
Overall (summary)	Never-smoker	41	6214	6.60	1 (ref)	1 (ref)
*N* = 1388	Past-smoker	73	5294	13.79	1.95 (1.32–2.90)	1.95 (1.32–2.90)
	Current-smoker	48	3078	15.59	3.95 (2.56–6.09)	3.90 (2.52–6.04)
By age group						
45–64 years	Never-smoker	7	4163	1.68	1 (ref)	1 (ref)
	Past-smoker	21	3510	5.98	3.60 (1.53–8.47)	3.62 (1.53–8.55)
	Current-smoker	28	2606	10.75	7.08 (3.08–16.28)	6.92 (2.98–16.04)
65–74 years	Never-smoker	10	1456	6.87	1 (ref)	1 (ref)
	Past-smoker	21	1294	16.23	2.49 (1.16–5.33)	2.53 (1.18–5.45)
	Current-smoker	12	381	31.50	4.86 (2.09–11.32)	5.51 (2.31–13.15)
≥75 years	Never-smoker	24	595	40.33	1 (ref)	1 (ref)
	Past-smoker	31	489	63.35	1.06 (0.58–1.94)	0.95 (0.50–1.78)
	Current-smoker	8	92	87.13	1.84 (0.80–4.20)	1.97 (0.83–4.66)

The Cox regression models for ‘Overall’ RR^1^ and RR^2^ violated the proportional hazard assumption when alpha = 0·05. As age is used for the underlying time scale of these models, the violation indicates an interaction between age and smoking status. Given the observed age differences in the smoking-mortality relationship, and the resulting proportional hazard assumption violation, a sensitivity analysis was undertaken with these models modified to have follow-up time as the underlying time scale, adjusted for 5-year age groups; see Table S3. Results of this sensitivity analysis were consistent with the results from the original ‘Overall’ models with the proportional hazards assumption violation, supporting the robustness of the original models. Given these consistent results, and because the original models allow finer adjustment for age, with age being one of the strongest predictors of mortality, we have retained the original models for the main results despite the proportional hazard violation.

RR^1^: adjusted for age as the underlying time variable and sex; RR^2^: additionally adjusted for education and remoteness. Rates are presented per 1000 person-years.

P-years, person-years; RR, relative risk; 95% CI, 95% confidence interval.

Results were not materially changed when: follow-up time was used as the underlying time scale (Supplementary Table S3, available as Supplementary data at *IJE* online); adjustment for alcohol intake was added (Supplementary Table S4); recent quitters were reclassified as current-smokers (Supplementary Table S5); or with sex or remoteness stratification (Supplementary Table S6).

**Figure 1:**
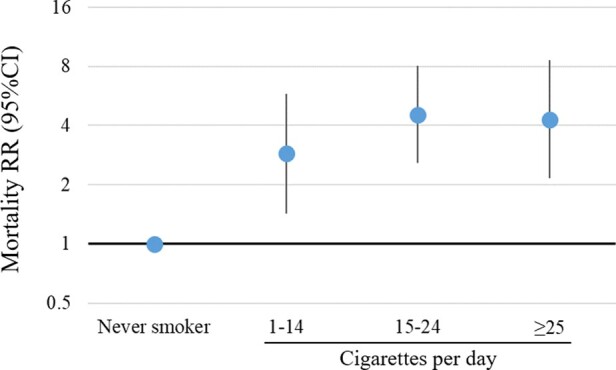
Relative risks of all-cause mortality among current-smokers relative to never-smokers, by smoking intensity. RR, relative risk. RR^2^: adjusted for age as the underlying time variable, sex, education and remoteness. *P*-trend for RR^2^ <0.01. To test for a trend in the relationship between smoking intensity and mortality, the fully adjusted model was re-run with smoking intensity as a continuous variable, with each category recoded to the median smoking intensity within that category. The Cox regression models for smoking intensity violated the proportional hazard assumption for the main exposure using the *P*-value threshold of 0.05. As age is used for the underlying time variable, violations of proportional hazards assumption are likely to be due to interaction with age

Compared with never-smokers, mortality RRs increased with increasing smoking intensity among current-smokers (*P*-trend < 0.01) and were 2.88 (1.43–5.80) with 1–14 cigarettes/day and 4.29 (2.15–8.57) with ≥25 cigarettes/day (Table 4; Figure 1).

**Table 4:** Absolute rates and relative risks of all-cause mortality among Aboriginal participants in the 45 and Up Study, by smoking intensity for current-smokers, and by age at cessation for past-smokers, relative to never-smokers

		Deaths	P-years	Crude rate	RR^1^ (95% CI)	RR^2^ (95% CI)
Smoking intensity, in current-smokers (cigarettes/day)^a^	Never-smoker	41	6214	6.60	1 (ref)	1 (ref)
*N* = 870	1–14	11	810	13.58	2.75 (1.38–5.47)	2.88 (1.43–5.80)
	15–24	22	1410	15.61	4.81 (2.76–8.38)	4.55 (2.58–8.02)
	≥25	12	757	15.85	4.47 (2.25–8.86)	4.29 (2.15–8.57)
Age at cessation (years), in past-smokers	Never-smoker	41	6214	6.60	1 (ref)	1 (ref)
*N* = 1283	Quit at age ≤44	21	2902	7.24	1.38 (0.80–2.38)	1.48 (0.85–2.57)
	Quit at age 45–54	21	1424	14.74	2.28 (1.33–3.90)	2.21 (1.29–3.80)
	Current-smoker	48	3078	15.59	4.03 (2.60–6.25)	3.98 (2.56–6.19)

Participants with missing values for smoking intensity or age at smoking cessation were excluded from regression using those variables. Past-smokers who quit smoking at age ≥55 years are excluded from regression using age at cessation. We could not reliably estimate the relation of later smoking cessation (≥55 years) to mortality, given that older smokers are likely to have quit due to illness. However, the vast majority of past-smokers in this cohort quit before age 55 years. The Cox regression models for smoking intensity RR^1^ and RR^2^ violated the proportional hazard assumption for the main exposure using the *P*-value threshold of 0.05. As age is used for the underlying time variable, violations of proportional hazards assumption are likely to be due to interaction with age.

RR^1^: adjusted for age as the underlying time variable and sex; RR^2^: additionally adjusted for education and remoteness. Rates are presented per 1000 person-years.

P-years, person-years; RR, relative risk; 95% CI, 95% confidence interval.

a P-trend for RR^2^ <0.01 (only tested for smoking intensity). To test for a trend in the relationship between smoking intensity and mortality, the fully adjusted model was re-run with smoking intensity as a continuous variable, with each category recoded to the median smoking intensity within that category.

**Figure 2:**
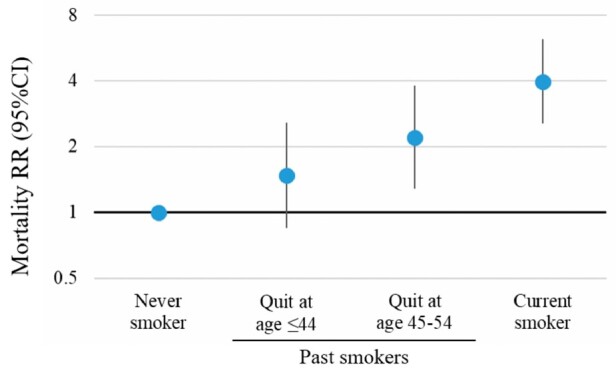
Relative risks of all-cause mortality among past-smokers, by age at cessation, and current-smokers relative to never-smokers. RR, relative risk. RR^2^: adjusted for age as the underlying time variable, sex, education and remoteness. Past smokers with missing values for age at smoking cessation, and past-smokers who quit smoking at age ≥55 years, are excluded

Compared with never-smokers, mortality risk was not significantly different for those who ceased smoking aged ≤44 (RR = 1.48; 0.85–2.57). Mortality RR for those ceasing smoking at age 45–54 was significantly higher than never-smokers (RR = 2.21; 1.29–3.80) (Table 4; Figure 2). When compared with current-smokers, mortality risk was significantly lower for past-smokers, overall (RR = 0.50; 0.34–0.74) and for those who quit at ≤44 years (RR = 0.37; 0.22–0.64) or 45–54 years (RR = 0.56; 0.33–0.95) (Supplementary Table S7, available as Supplementary data at *IJE* online).

Of deaths in the cohort with underlying cause recorded, 13% were from lung cancer, 19% other cancers established as caused by smoking, 19% circulatory disease, and 7% respiratory disease (Table 5). The underlying cause of death was from a condition made more likely by smoking for 87% of deaths among current-smokers, and 79% for past-smokers and never-smokers.

**Table 5:** Underlying cause of death among Aboriginal current, past and never-smokers in the 45 and Up Study

Underlying cause of death (CoD)	% of deaths due to each cause			
	Smoking status			Total
	Current	Past	Never	
CoD established as causally linked to smoking				
Lung cancer	23	≤13	—	13
Other cancers established as caused by smoking	23	15	21	19
Diseases of the circulatory system established as caused by smoking	18	19	18	19
Diseases of the respiratory system established as caused by smoking	—	≤13	—	7
Diabetes	—	—	—	4
CoD likely to be, but not established as, causally linked to smoking	21	18	24	20
Total deaths due to conditions made appreciably more probable by smoking	87	79	79	81
CoD unlikely to be causally linked to smoking	13	21	21	19

Restricted to deaths where the cause of death was recorded. Cause of death was not recorded for 17% of deaths in the sample. Numbers of events are not provided to protect confidentiality; —represents cells containing fewer than five events, with data not shown, to protect confidentiality. See Table S2 for details on the classification of ICD-10AM codes.

CoD, cause of death.

**Figure 3:**
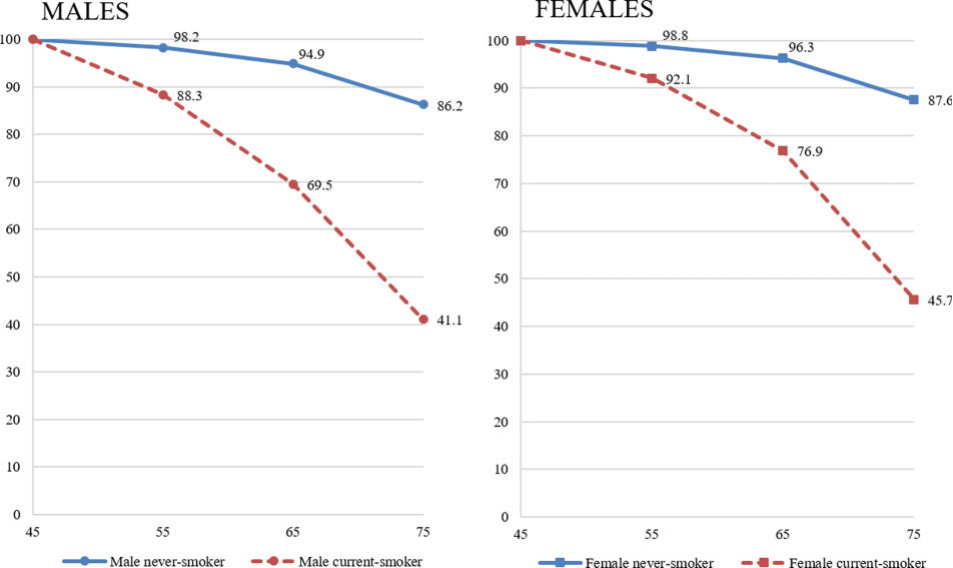
Illustration of survival from age 45 to 75 years for smokers and never-smokers in the Aboriginal and Torres Strait Islander population, by sex. This figure presents the estimated percent of never-smokers and current-smokers surviving from age 45 to age 55, 65 and 75 years, as an illustration of the absolute effects of the observed relative risks. RR^2^s, the fully adjusted risk ratios (R) estimated in this study were used, along with 2018 mortality rates for the Aboriginal and Torres Strait Islander population (M), and 2008 national smoking prevalence (p) from other sources* to estimate absolute mortality rates by smoking status (k; never, past and current) and age group (45–54, 55–64 and 65–74 years), separately for males and females. Mortality rate for the reference group (never-smokers) M_0_ was estimated as M/(1+∑_k_(R_k_-1)p_k_). Mortality for k^th^ group, M_k_, was estimated as R_k_M/(1+∑_k_(R_k_-1)p_k_). From these rates, cumulative risks of death for current- and never-smokers at age x (55, 65 or 75 years) from age 45 were estimated by $1-e^{-10\sum_{i=45-54}^{x}m_{k}}$ where m_k_ is current- or never-smoker mortality rate for age group i. The percentage of each group surviving was estimated as 1 minus the cumulative risk of death. The formula used to derive cumulative risk makes the following assumptions: the population is closed, there is no competing risk and the number of events at each event time is a small proportion of the number at risk. *Age-group specific deaths data are only available for five of the eight states/territories in Australia: New South Wales, Queensland, South Australia, Western Australia and the Northern Territory, due to data quality limitations. National deaths in each age-sex category were estimated based on data extracted from the Australian Bureau of Statistics’ ABS.Stat [http://stat.data.abs.gov.au/], as in Table 6. National Aboriginal and Torres Strait Islander smoking prevalence estimates from 2008 were generated through analysis of microdata from the 2008 National Aboriginal and Torres Strait Islander Social Survey (NATSISS), Expanded Confidentialised Unit Record File (CURF). This approach was developed based on methods from Liu *et al*.^18^ and Schouten *et al*.^19^

Applying the RRs from this study, hypothetically, 86.2% of male and 87.6% female never-smokers would survive to age 75, compared with 41.1% of male and 45.7% female current-smokers (Figure 3). Average survival at ages 55 and 65 for never-smokers, compared with current-smokers, was 14–16 years longer for males and 13 years longer for females.

Half (50.3%) of all contemporary deaths among Aboriginal and Torres Strait Islander adults aged ≥45 years are caused by smoking, if 90% of the excess deaths among current- and past-smokers are due to their smoking (Table 6). Over the past decade, >10 000 deaths among Aboriginal and Torres Strait Islander adults aged ≥45 were caused by smoking. The SAF is 44.8% (9500 deaths) if 80% of the excess deaths are smoking-attributable, and 55.9% (11 900 deaths) if all excess deaths are smoking-attributable. The SAF was 54.4% (6000 deaths) for males ≥45 years, and 46.0% (4700 deaths) for females ≥45 years.

**Table 6:** Smoking-attributable fraction (SAF) during cohort follow up, and smoking-attributable deaths 2009-2018, for adults aged ≥45 years

	2008 smoking status (proportion)			Past-smoker: sex-combined mortality RR			Current-smoker: sex-combined RR				Smoking-attributable fraction (SAF)^b^			National smoking-attributable deaths 2009-18^b^		
	Current	Past	Never	RR	LCI	UCI	RR	LCI	UCI	National total deaths 2009-2018^a^	Estimate (∝=0.90)	Lower bound (∝=0.80)	Upper bound (∝=1.00)	Estimate (∝=0.90)	Lower bound (∝=0.80)	Upper bound (∝=1.00)
Males																
45–64 years	0.462	0.338	0.201	3.62	1.53	8.55	6.92	2.98	16.04	2953	70.5	62.7	78.4	2083	1851	2314
65–74 years	0.262	0.520	0.218	2.53	1.18	5.45	5.51	2.36	13.15	3105	59.8	53.1	66.4	1856	1650	2062
≥65 years	0.120	0.721	0.159	0.95	0.50	1.78	1.97	0.83	4.66	5052	7.9	7.0	8.8	400	355	444
						Male deaths at age ≥45 years				11 110	54.4	48.3	60.4	6042	5371	6714
						Male deaths at all ages				15 890	38.0	33.8	42.3	6042	5371	6714
Females																
45–64 years	0.437	0.254	0.309	3.62	1.53	8.55	6.92	2.98	16.04	1993	68.8	61.2	76.5	1372	1219	1524
65–74 years	0.208	0.264	0.528	2.53	1.18	5.45	5.51	2.36	13.15	2484	51.6	45.8	57.3	1281	1139	1423
≥65 years	0.166	0.401	0.433	0.95	0.50	1.78	1.97	0.83	4.66	5738	11.1	9.9	12.4	638	567	709
						Female deaths at age ≥45 years				10 215	46.0	40.8	51.1	4694	4173	5216
						Female deaths at all ages				13 181	35.6	31.7	39.6	4694	4173	5216
Persons																
						Total deaths at age ≥45 years				21 325	50.3	44.8	55.9	10 737	9544	11 930
						Total deaths at all ages				29 071	36.9	32.8	41.0	10 737	9544	11 930

Current smoking prevalence includes daily and non-daily smokers; the vast majority of current-smokers are daily smokers.

RR, relative risk; LCI, lower confidence interval; UCI, upper confidence interval; SAF, smoking-attributable fraction.

a National estimates of annual total deaths by sex were extracted from the Australian Bureau of Statistics’ ABS.Stat [http://stat.data.abs.gov.au/]. Age-group-specific deaths data are only available for five of the eight states/territories in Australia (New South Wales, Queensland, South Australia, Western Australia and the Northern Territory) due to data quality limitations. To estimate national age-sex group deaths, we applied a scale factor (total number of deaths by sex/five state-territory deaths by sex) to the number of deaths in each age-sex group in the five state-territory data. The mortality statistics used may underestimate deaths in this population, due to potential misclassification of Aboriginal and/or Torres Strait Islander deaths as non-Aboriginal, and lags in death registration. It has been estimated that over 2001-15, 13.5% of all male and 13.9% of all female Aboriginal and Torres Strait Islander deaths were misclassified as non-Indigenous deaths.^20^ Misclassification was particularly common among those aged ≥65 years (19.3% and 17.7%, respectively), but still high among those aged 45–64 years (11.9% and 11.0%, respectively). If this rate of misclassification was consistent over the 2009-18 period, we would have underestimated deaths by 15.0% (missing 3204 deaths ≥45 years) (Table S12).

b According to assumptions that 90%, 80% and 100% of excess deaths among smokers are smoking-attributable. We calculated the smoking-attributable fraction (SAF) using the prevalence-based method, for the age groups 45-64, 65–74, and ≥75 years: SAF(%)=100x[P_p_(RR_p_-1)+P_c_(RR_c_-1)]/[P_p_(RR_p_-1)+P_c_(RR_c_-1)+1]. Here, P_p_ and P_c_ are the prevalence of past and current smoking, respectively, and RR_p_ and RR_c_ are the RRs for mortality among past- and current-smokers, respectively, compared with never-smokers. The smoking-attributable fraction is calculated using sex-combined RR^2^ results for participants aged 45–64, 65–74 and ≥75 years, adjusted for age as the underlying time variable, sex, education and remoteness.

Assuming no deaths <45 years are smoking-attributable, the SAF for all ages is 36.9%. If we assume that 35–44 year-olds experience the same smoking-attributable RR as 45–64-year-olds, the SAF is around 70% for this age group, and 44.5% for all ages (Supplementary Table S8, available as Supplementary data at *IJE* online). The SAFs were similar using 2018/19 and 2008 smoking prevalences (Supplementary Table S9, available as Supplementary data at *IJE* online).

## Discussion

Around half of all contemporary deaths of Aboriginal and Torres Strait Islander adults aged ≥45 years are caused by smoking, according to this study. Over the past decade, this amounts to >10 000 preventable premature deaths. Never-smokers were around twice as likely to survive to age 75, and had over an extra decade of life expectancy, compared with current-smokers. These findings highlight the magnitude of smoking-related harms, and the urgent need to prevent smoking initiation and to support Aboriginal and Torres Strait Islander smokers to quit.

This is the first study to directly quantify smoking-attributable mortality in the Aboriginal and/or Torres Strait Islander population and the first study in an Indigenous population internationally able to reliably quantify this relationship. The only previous findings internationally, that we were able to locate, were from analysis of linked data among Māori people in New Zealand and were not able to account for pre-existing disease.^9^^,^^21^ In this cohort of Aboriginal adults aged ≥45 years, the risk of dying in current-smokers was 4-fold and past-smokers was almost double that of never-smokers. Most excess deaths among current- and past-smokers (87% and 79%) were due to conditions where risk is increased by smoking. Risk increased with increasing smoking intensity, and was substantially reduced by smoking cessation.

The SAF represents the percentage of premature deaths that could have been averted if past risk exposure was eliminated, i.e. if all current- and past-smokers had never smoked, demonstrating the enormous potential for health gain through reducing smoking prevalence The high SAF observed reflects the strong mortality risk associated with smoking, and the high prevalence in the population. Only 26.3% of adults aged ≥45 nationally were never-smokers in 2018/19.

Estimates of near-future SAF were similar to the SAF from 2009–18, reflecting that although smoking prevalence in the total Aboriginal and Torres Strait Islander population has decreased substantially in the past decade, smoking prevalence within older age groups (≥45 years) has remained relatively stable.^13^ If we assume that 35–44 year olds experience the same RR as 45–65 year olds, around two-thirds of deaths among 35–74 year old males (68.1%) and females (63.9%) are caused by smoking (Supplementary Table S8, available as Supplementary data at IJE online). In comparison, peak estimated SAFs in the total Australian population aged 35–69 was 39% for males (around 1975), and 18% for females (around 1995).^22^

We conservatively estimate that smoking causes over a third (36.9%) of all deaths in the Aboriginal and Torres Strait Islander population; the estimate increases to 44.5% if 35–44 year olds experience the same mortality RRs as 45–64 year olds. The harms of smoking, and the contribution to population-level mortality, may be underestimated by not accounting for second-hand smoke (SHS) exposure. Aboriginal and Torres Strait Islander peoples have higher SHS exposure than the non-Indigenous population.^2^^,^^23^ Exposure to SHS at baseline was high in this sample, among both smokers and never-smokers (Supplementary Table S10, available as Supplementary data at *IJE* online). Exclusion of participants with SHS exposure at baseline did not materially change RRs, but led to considerably greater uncertainty (data not shown). Our conservative SAF estimate is almost double the 2003 Burden of Disease estimate (20.0%).^24^ These estimates are not directly comparable given methodology differences, but it is apparent the full impact of smoking on Aboriginal and Torres Strait Islander health has not been recognized.

The SAF was high for males and females ≥45 years, at 54.4% and 46.0% respectively, contrasting with the global burden where over 75% of SAM is among males.^25^ The high smoking prevalence among Aboriginal and Torres Strait Islander, as well as Māori, females has been traced back to colonization, and practices that led to widespread tobacco use by males and females.^1^^,^^2^^,^^9^

We observed substantial age heterogeneity in the smoking-mortality relationship, as has been observed in other populations.^12^ Mortality risk was over 5-fold for current- versus never-smokers aged 45–74, but attenuated among adults ≥75 years. This may reflect the high base mortality rate in the older age group, and competing (non-smoking-related) causes of death. Of all Aboriginal and Torres Strait Islander current- and past-smokers aged ≥45 years nationally in 2018/19, the vast majority (97.3%, 69 547/71 484 current; 91.3%, 55 128/60 399 past) are aged <75 years.^6^

Indigenous populations are diverse and although smoking will have a major impact on their mortality, some variation in RR is expected. The mortality RRs observed among current-smokers means that smoking is likely to have caused up to three-quarters of the deaths in this group. The magnitude of this RR is consistent with evidence from the total Australian population, and international cohorts, which demonstrate mortality risks around 3 in current- versus never-smokers.^8^^,^^26^^,^^27^ In contrast, fully adjusted Māori RRs were 1.28 (1.14–1.44) for men and 1.38 (1.21–1.58) for women aged 25–74 in analysis of linked census and mortality data from New Zealand, 2006–11.^9^ The apparent discrepancy may be explained by the inclusion of participants with pre-existing disease in the linked census study, potentially biasing RRs towards the null; this ‘sick quitter’ effect is likely to differentially affect Māori, given higher disease prevalence. Exclusion of participants with baseline cardiovascular disease and cancer, in line with international best practice,^12^^,^^26^^,^^27^ is a strength of the current study.

Participants smoking 1–14 cigarettes per day (mean: 10) had a 3-fold mortality risk compared with never-smokers, consistent with previous findings, and increasing recognition of the harms of so-called ‘light smoking’.^8^^,^^12^^,^^26^ The mortality risk for people who quit smoking before age 45 did not differ significantly from that of never-smokers. Those who quit at age 45–54 years had a mortality risk half that of current-smokers and double that of never-smokers. This demonstrates the benefits of quitting at all ages examined, compared with continuing to smoke, consistent with other findings.^8^^,^^12^^,^^26^

Within the Aboriginal and Torres Strait Islander population, there are generally high levels of knowledge of smoking-related disease, although there is some evidence of inconsistency in the level of awareness of harms.^1^ These results provide community-specific evidence of risk that can inform communication and behaviour change campaigns highly salient to Aboriginal and Torres Strait Islander peoples. We note that lack of knowledge about smoking’s harms is not a major barrier to quitting.^28^^,^^29^ However, focusing on the potential reversal of risk through early cessation could help counteract fatalism about health, where this occurs.^28^^,^^30^ Understanding the extent of smoking’s harms, and the benefits of quitting, should guide policy and funding decisions that support Aboriginal and Torres Strait Islander communities to reduce smoking prevalence.

Our estimates provide a ‘plausible range’ of current/future deaths caused by smoking. The accuracy of the SAF estimation depends on multiple inputs—i.e. the RR, smoking prevalence and mortality estimates—each with an associated level of uncertainty.^15^ The findings on SAM should be interpreted accordingly. Similarly, the survival curves are subject to uncertainty and are presented for illustrative purposes.

The Aboriginal cohort within the 45 and Up Study is not representative of all Aboriginal and Torres Strait Islander adults. The prevalence of never-smoking in the cohort (41.9%) is higher than in the total population aged ≥45 (27.8% in 2008), which is typical of the ‘healthy cohort effect’. However, within-population estimates (i.e. the RRs in this study), are understood to be generalizable beyond the cohort.^31^ The cohort’s base mortality rate aligns with that of the NSW and national Aboriginal and Torres Strait Islander population (Supplementary Table S11, available as Supplementary data at *IJE* online).

The Aboriginal cohort within the 45 and Up Study is one of the largest sources of linked survey and mortality data about Aboriginal and Torres Strait Islander adults. However, the sample is relatively small in absolute terms, resulting in large CIs and limited ability to adjust for potential confounders, with potential to influence RR and SAF estimates. The magnitude of any under- or over-estimation is likely to be small^15^^,^^26^; for example, additional adjustment for alcohol intake did not materially change results. There are slight differences in the definition of smoking status between the 45 and Up Study data used to calculate mortality risk associated with smoking and the ABS surveys used to estimate smoking prevalence (Supplementary Table S12, available as Supplementary data at *IJE* online).^15^

We estimated the causal contribution of smoking to mortality by applying a scale factor to the SAF, using values that plausibly reflect the proportion of excess deaths among current-smokers and past-smokers attributable to smoking. Given the lack of population-specific evidence, we used estimates from pooled data from almost 1 million US men and women aged ≥55 years followed over 2000–11.^11^ In that study, 83% of excess deaths among current-smokers were from conditions established as causally linked to smoking, with an additional 10% from conditions likely to be caused by smoking (total 93%). Corresponding figures for past-smokers were 80% and 90%.^11^ It is unknown if these figures are accurate for the current cohort. We have quantified uncertainty by providing plausible lower and upper bound estimates. Our calculations of SAM did not take into account smoking intensity or duration, or age at cessation for past-smokers.

In the total 45 and Up Study cohort, there is evidence of highly accurate probabilistic matching (targets of ≤0.5% for false-negatives and estimate of 0.5% for false-positives).^32^ However, the accuracy for Aboriginal participants is unknown. Inaccurate matching could result in under- or over-estimation of SAM.^15^

Our analysis of the relation of smoking to mortality was based on self-reported smoking status at baseline. Participants’ smoking status and/or intensity may have changed during follow-up. Resurvey of a sample of 60 404 participants from the total 45 and Up Study cohort at mean 3.3 years post-baseline found that <2% of never- and past-smokers at baseline were current-smokers at follow-up, and that one-third of current-smokers at baseline were no longer smoking at follow-up.^8^ If a similar pattern is observed within the Aboriginal participants, the estimated RRs for current-smokers are likely to be conservative.

These findings provide a clear case for sustained and increased outcome-focused action in tobacco control, prioritizing the Aboriginal and Torres Strait Islander population and supported by total population approaches. Continuing reductions in youth uptake, increasing cessation (at all ages) and reducing SHS exposure should be national priorities. The majority of Aboriginal and Torres Strait Islander smokers want to quit.^33^ However, the legacies of historical policies, entrenched dependence, a history of comprehensive and pervasive marketing and a predatory tobacco industry have undermined choice.^2^^,^^3^^,^^5^ For Indigenous peoples, there is an urgent need to empower an informed choice to be smoke-free, addressing dependence in context. Our findings demonstrate the clear need for high-quality population-specific data for Indigenous populations globally.

Linked participant data from the 45 and Up Study are accessible to researchers through application; processes and requirements are detailed at: [https://www.saxinstitute.org.au/our-work/45-up-study/for-researchers/].

## Supplementary data

Supplementary data are available at *IJE* online.

## Funding

K.T., E.B., R.L. are supported by the National Health and Medical Research Council of Australia [K.T.: 1156276, R.L.: 1122273, E.B.: 1136128]. S.W., G.B. are supported by VicHealth.

## Supplementary Material

dyaa274_Supplementary_Data
