# Supplementary material for: Tobacco smoking and mortality among Aboriginal and Torres Strait Islander adults in Australia
Source: Int J Epidemiol. 2021 Jan 25;50(3):942–54. doi: 10.1093/ije/dyaa274 (PMC8271186; doi:10.1093/ije/dyaa274)

**SUPPLEMENTARY MATERIAL**

**Table S1. Definition of variables in the 45 and Up Study, and for national smoking prevalence estimates from ABS health and social surveys**

| **45 and Up Study** | |
| --- | --- |
| Smoking status | Smoking status was determined according to the responses to: “Have you ever been a regular smoker? If ‘Yes’, how old were you when you started smoking regularly? Are you a smoker now? If not, how old were you when you stopped smoking regularly? About how much do you/did you smoke on average each day?” Those who reported that they had never been a regular smoker were coded as never-smokers. Those who reported that they had ever been, and were currently, a regular smoker were coded as current smokers. Those who reported that they had ever been, but were no longer, a regular smoker were coded as past smokers. |
| Age at smoking initiation | Among current and past smokers, age at commencing smoking was categorised as <16, 16-20, or ≥21 years. |
| Smoking intensity | Smoking intensity was categorised as 1-14, 15-24, or ≥25 cigarettes/day. |
| Age at smoking cessation | Among past smokers, age at smoking cessation was categorised as <30, 30-44, 45-54, or ≥55 years. |
| Smoking duration | Smoking duration was defined as years of smoking until cessation (for past smokers) or until baseline (for current smokers), and categorised as <25, 25-39, or ≥40 years. |
| Second-hand smoke (SHS) exposure | Current second-hand smoke exposure was determined based on participants’ estimation of the number of hours per week of exposure to someone else’s tobacco smoke, in the home, and in other places (e.g. work, going out, cars). Participants were dichotomised as having any or no second-hand smoke exposure at home, and in other places, and were also categorised according to total level of average daily exposure (no exposure, >0 to <1 hour per day, or ≥1 hour per day). Data were only available on current—not lifetime—second-hand smoke exposure, so second-hand exposure was not accounted for in the calculation of RRs. |
| Remoteness | Remoteness was categorised as major city, inner regional, outer regional/remote (including very remote), or missing, based on the mean Accessibility Remoteness Index of Australia (ARIA+) for the participant’s postcode. |
| Highest education qualification | Education was categorised as no school certificate; certificate, diploma, or trade/apprenticeship; university degree or higher; or, missing. |
| Annual household income | Annual household income from all sources (including benefits, pensions, superannuation) was categorised as <$20 000, $20 000-$39 999, $40 000-$69 999, ≥$70 000, or missing. |
| Functional limitation | Functional limitation was defined according to the Medical Outcomes Study Physical Functioning (MOS-PF) scale, which indicates the extent to which a participant’s health limits their daily functional activities. Participants were categorised as having no limitation (score ≥75), or any limitation (score <75). |
| BMI | BMI was calculated based on participants’ estimation of their own weight and height, calculated as weight (kg) divided by the square of height (m^2^)· BMI was categorised as 15 to 19·9 kg/m^2^, 20 to 24·9 kg/m^2^, 25 to 29·9 kg/m^2^, 30 to 50 kg/m^2^· |
| Alcohol | Alcohol intake was determined based on participants’ estimation of how many alcohol drinks they consume each week (“one drink = one glass of wine, middy of beer or nip of spirits”). Intake was categorised as none, 1-14 drinks, 15 or more drinks, or missing. |
| Private health insurance | Private health insurance status was categorised Hospital/DVA insurance (private health insurance – with extras, private health insurance – without extras, Department of Veterans’ Affairs (DVA) white or gold card holder, health care concession card holder), or no private health insurance. |
| **ABS health and social surveys (2008 and 2018/19)** | |
| Smoking status | Current smoker is defined as a daily or less frequent smoker; ex-smoker includes all who are not a current smoker but had ever smoked daily or had smoked cigarettes ≥100 times or other tobacco products ≥20 times; never-smoker is defined as having smoked cigarettes fewer than 100 times or other tobacco products fewer than 20 times. |

RR = Relative Risk. ABS = Australian Bureau of Statistics.

**Table S2. Broad categorisation of causes of death, according to ICD-10AM codes**

| **Category** | **Included ICD-10AM codes** |
| --- | --- |
| **Conditions established as causally linked to smoking** |  |
| Lung cancer | C33-C34 |
| Other cancers established as caused by smoking | C00–C14, C15, C16, C18–C20, C22, C25, C32, C53, C64-66, C67, C92.0 |
| Diseases of the circulatory system established as caused by smoking | I03–I09, I20-I25, I26-I51, I60–I69, I70, I71, I72–I78 |
| Diseases of the respiratory system established as caused by smoking | A16–A19, J10-18, J40–J44 |
| Diabetes | E10–E14 |
| **Additional conditions associated with smoking** |  |
| Other cancers likely to be caused by smoking | C17, C21, C23, C24, C26, C30, C31, C37–C41, C45, C47–C49, C50, C51, C52, C54, C55, C60, C61, C63, C68, C70–C70.1, C72, C74, C76–C79, C81.0–C81.3, C96, C97, D00–D48.7, D48.9 |
| Other diseases likely to be caused by smoking | A00–A15, A20–B99, H00–H95, I10, I11, I15, J00-J9, J19-J39, J45–J99, K00–K52.9, K55–K67, K70, K74, K80–K93, L00–L99, M00–M99, N17-N19, O00–R98, R99 |
| **Other conditions unlikely to be causally linked to smoking** |  |
| Other cancers unlikely to be caused by smoking | All cancers not listed above |
| External causes | U50-Y98 |
| Other diseases unlikely to be caused by smoking | Any other causes not listed above |
|  |  |
| **Deaths due to causes where risk is increased by smoking** | A00–B99, C00–C20, C22-C26, C30-C34, C37–C41, C45, C47–C55, C60, C61, C63-C68, C70–C70.1, C72, C74, C76–C79, C81.0–C81.3, C92.0, C96, C97, D00–D48.7, D48.9, E10–E14, H00–H95, I03–I11, I15, I20-I51, I60–I78, J00-J99, K00–K52.9, K55–K67, K70, K74, K80–K93, L00–L99, M00–M99, N17-N19, O00–R98, R99 |

ICD-10AM = International Statistical Classification of Diseases and Related Health Problems 10, Australian Modification.

These categories were based on those presented in Supplementary Appendix C of the publication: *Carter, B. D., et al. (2015). "Smoking and mortality—beyond established causes." New England Journal of Medicine 372(7): 631-640.* Carter et al. categorised causes of death (based on ICD-10) as established as attributable to smoking, linked to smoking (but not yet established as caused by smoking), and unlikely to be caused by smoking. This table presents ICD-10AM codes aligned with Carter’s classification, with minor modifications.

**Table S3. Absolute rates and relative risks of all-cause mortality among Aboriginal current and past smokers in the 45 and Up Study, relative to never-smokers, overall using follow-up time (rather than age) as the underlying time scale**

|  | | **Deaths** | **P-years** | **Crude rate** | **RR^1^ (95%CI)** | **RR^2^ (95%CI)** |
| --- | --- | --- | --- | --- | --- | --- |
| **Overall** |  |  |  |  |  |  |
| N=1,388 | Never smoker | 41 | 6214 | 6·60 | 1 (ref) | 1 (ref) |
|  | Past smoker | 73 | 5294 | 13·79 | 1·95 (1·31-2·9) | 1·95 (1·31-2·90) |
|  | Current smoker | 48 | 3078 | 15·59 | 3·71 (2·40-5·73) | 3·64 (2·34-5·64) |

P-years = person-years. RR = Relative Risk. 95%CI = 95% Confidence Interval.

RR^1^: adjusted for sex and 5-year age-groups (follow-up time as the underlying time scale). RR^2^: additionally adjusted for education and remoteness. Rates are presented per 1000 person-years.

**Table S4. Absolute rates and relative risks of all-cause mortality among Aboriginal current and past smokers in the 45 and Up Study, relative to never-smokers, with additional adjustment for alcohol intake**

|  |  | **Deaths** | **P-years** | **Crude rate** | **RR^1^ (95%CI)** | **RR^2^ (95%CI)** | **RR^3^ (95%CI)** |
| --- | --- | --- | --- | --- | --- | --- | --- |
| **Overall** |  |  |  |  |  |  |  |
| N=1,388 | Never smoker | 41 | 6214 | 6·60 | 1 (ref) | 1 (ref) | 1 (ref) |
|  | Past smoker | 73 | 5294 | 13·79 | 1·95 (1·32-2·90) | 1·95 (1·32-2·90) | 2·07 (1·38-3·10) |
|  | Current smoker | 48 | 3078 | 15·59 | 3·95 (2·56-6·09) | 3·90 (2·52-6·04) | 4·07 (2·62-6·33) |

P-years = person-years. RR = Relative Risk. 95%CI = 95% Confidence Interval.

RR^1^: adjusted for age as the underlying time variable and sex. RR^2^: additionally adjusted for education and remoteness. RR^3^: additionally adjusted for alcohol intake. Rates are presented per 1000 person-years.

**Table S5. Absolute rates and relative risks of all-cause mortality among Aboriginal current and past smokers in the 45 and Up Study, relative to never-smokers, with past smokers reclassified as current smokers if they had quit within three years of the baseline survey**

|  |  | **Deaths** | **P-years** | **Crude rate** | **RR^1^ (95%CI)** | **RR^2^ (95%CI)** |
| --- | --- | --- | --- | --- | --- | --- |
| **Overall** |  |  |  |  |  |  |
| N=1,388 | Never smoker | 41 | 6214 | 6·60 | 1 (ref) | 1 (ref) |
|  | Past smoker | 64 | 4651 | 13·76 | 1·91 (1·27-2·85) | 1·89 (1·26-2·84) |
|  | Current smoker | 57 | 3722 | 15·32 | 3·62 (2·38-5·50) | 3·64 (2·39-5·54) |

P-years = person-years. RR = Relative Risk. 95%CI = 95% Confidence Interval.

RR^1^: adjusted for age as the underlying time variable and sex. RR^2^: additionally adjusted for education and remoteness. Rates are presented per 1000 person-years.

The Cox regression model for “Overall” RR^1^ and RR^2^ violated the proportional hazard assumption for the main exposure using the p-value threshold of 0·05. As age is used for the underlying time variable, violations of proportional hazards assumption are likely to be due to interaction with age.

**Table S6. Absolute rates and relative risks of all-cause mortality among Aboriginal current and past smokers, relative to never-smokers, in the 45 and Up Study by sex and remoteness**

|  |  | **Deaths** | **P-years** | **Crude rate** | **RR^1^ (95%CI)** | **RR^2^ (95%CI)** |
| --- | --- | --- | --- | --- | --- | --- |
| **SEX** |  |  |  |  |  |  |
| **Males** |  |  |  |  |  |  |
| N=603 | Never smoker | 13 | 2331 | 5·58 | 1 (ref) | 1 (ref) |
|  | Past smoker | 44 | 2587 | 17·01 | 1·99 (1·06-3·75) | 1·96 (1·04-3·68) |
|  | Current smoker | 29 | 1348 | 21·52 | 5·16 (2·66-10·03) | 4·90 (2·49-9·63) |
| **Females** |  |  |  |  |  |  |
| N=785 | Never smoker | 28 | 3883 | 7·21 | 1 (ref) | 1 (ref) |
|  | Past smoker | 29 | 2706 | 10·71 | 1·92 (1·13-3·25) | 1·89 (1·11-3·21) |
|  | Current smoker | 19 | 1731 | 10·98 | 2·85 (1·55-5·23) | 2·83 (1·53-5·21) |
|  |  |  |  |  |  |  |
| **REMOTENESS** |  |  |  |  |  |  |
| **Major cities** |  |  |  |  |  |  |
|  | Never smoker | 20 | 2334 | 8·57 | 1 (ref) | 1 (ref) |
|  | Past smoker | 27 | 2066 | 13·07 | 1·49 (0·81-2·75) | 1·49 (0·81-2·77) |
|  | Current smoker | 16 | 1098 | 14·58 | 3·50 (1·75-7·01) | 3·59 (1·78-7·23) |
| **Non-major cities** |  |  |  |  |  |  |
|  | Never smoker | 21 | 3749 | 5·60 | 1 (ref) | 1 (ref) |
|  | Past smoker | 45 | 3133 | 14·36 | 2·12 (1·25-3·60) | 2·14 (1·25-3·65) |
|  | Current smoker | 31 | 1965 | 15·78 | 4·24 (2·40-7·51) | 4·26 (2·39-7·58) |

P-years = person-years. RR = Relative Risk. 95%CI = 95% Confidence Interval.

RR^1^: adjusted for age as the underlying time variable and sex. RR^2^: additionally adjusted for education and remoteness. Rates are presented per 1000 person-years. Participants missing data on remoteness were excluded from the regression for remoteness.

The Cox regression models for Males RR^1^, Females RR^1^ and RR^2^, and Major cities RR^1^ violated the proportional hazard assumption for the main exposure using the p-value threshold of 0·05. As age is used for the underlying time variable, violations of proportional hazards assumption are likely to be due to interaction between age and smoking status.

**Table S7. Absolute rates and relative risks of all-cause mortality among Aboriginal never, current, and past smokers in the 45 and Up Study, overall and by smoking intensity and age at cessation, with modified reference group**

|  | | **Deaths** | **P-years** | **Crude rate** | **RR^1^ (95%CI)** | **RR^2^ (95%CI)** |
| --- | --- | --- | --- | --- | --- | --- |
| **Total** | | 162 | 14 586 | 11·11 | **--** | **--** |
| **Overall** | Never smoker | 41 | 6214 | 6·60 | 0·25 (0·16-0·39) | 0·26 (0·17-0·40) |
| N=1,388 | Past smoker | 73 | 5294 | 13·79 | 0·49 (0·34-0·72) | 0·50 (0·34-0·74) |
|  | Current smoker | 48 | 3078 | 15·59 | 1 (ref) | 1 (ref) |
|  |  |  |  |  |  |  |
| **Smoking intensity, in current smokers (cigarettes/day)** | Never smoker | 41 | 6214 | 6·60 | 0·22 (0·11-0·44) | 0·23 (0·12-0·46) |
| N=870 | 1-14 | 11 | 810 | 13·58 | 0·62 (0·26-1·46) | 0·67 (0·28-1·62) |
|  | 15-24 | 22 | 1410 | 15·61 | 1·08 (0·53-2·19) | 1·06 (0·52-2·17) |
|  | ≥25 | 12 | 757 | 15·85 | 1 (ref) | 1 (ref) |
|  |  |  |  |  |  |  |
| **Age at cessation (years), in past smokers** | Never smoker | 41 | 6214 | 6·60 | 0·25 (0·16-0·39) | 0·25 (0·16-0·39) |
| N=1,283 | Quit at age ≤44 | 21 | 2902 | 7·24 | 0·34 (0·20-0·58) | 0·37 (0·22-0·64) |
|  | Quit at age 45-<55 | 21 | 1424 | 14·74 | 0·57 (0·33-0·96) | 0·56 (0·33-0·95) |
|  | Current smoker | 48 | 3078 | 15·59 | 1 (ref) | 1 (ref) |

P-years = person-years. RR = Relative Risk. 95%CI = 95% Confidence Interval.

Participants with missing values for smoking intensity or age at smoking cessation were excluded from regression using those variables. Past smokers who quit smoking at age ≥55 years are excluded from regression using age at cessation.

RR^1^: adjusted for age as the underlying time variable and sex. RR^2^: additionally adjusted for education and remoteness. Rates are presented per 1000 person-years.

**Table S8. Smoking attributable fraction during cohort follow up, and smoking-attributable deaths 2009-2018, for adults aged ≥35 years, if the 35-44 year age group experiences the same mortality RRs as the 45-64 year age group**

|  | **2008 smoking status (proportion)** | | |  | **Past smoker:**  **sex-combined mortality RR** | | |  | **Current smoker: sex-combined RR** | | |  | **National total deaths 2009-2018^a^** |  | **Smoking Attributable Fraction^b^** | | |  | **National smoking-attributable deaths 2009-2018^b^** | | |
| --- | --- | --- | --- | --- | --- | --- | --- | --- | --- | --- | --- | --- | --- | --- | --- | --- | --- | --- | --- | --- | --- |
|  | **Current** | **Past** | **Never** |  | **RR** | **LCI** | **UCI** |  | **RR** | **LCI** | **UCI** |  |  |  | **Estimate (∝=0·90)** | **Lower bound (∝=0·80)** | **Upper bound (∝=1·00)** |  | **Estimate (∝=0·90)** | **Lower bound (∝=0·80)** | **Upper bound (∝=1·00)** |
| **Males** |  |  |  |  |  |  |  |  |  |  |  |  |  |  |  |  |  |  |  |  |  |
| 35-44 years | 0·589 | 0·183 | 0·228 |  | 3·62 | 1·53 | 8·55 |  | 6·92 | 2·98 | 16·04 |  | 1903 |  | 71·9 | 63·9 | 79·9 |  | 1368 | 1216 | 1520 |
| 45-64 years | 0·462 | 0·338 | 0·201 |  | 3·62 | 1·53 | 8·55 |  | 6·92 | 2·98 | 16·04 |  | 2953 |  | 70·5 | 62·7 | 78·4 |  | 2083 | 1851 | 2314 |
| 65-74 years | 0·262 | 0·520 | 0·218 |  | 2·53 | 1·18 | 5·45 |  | 5·51 | 2·36 | 13·15 |  | 3105 |  | 59·8 | 53·1 | 66·4 |  | 1856 | 1650 | 2062 |
| ≥65 years | 0·120 | 0·721 | 0·159 |  | 0·95 | 0·50 | 1·78 |  | 1·97 | 0·83 | 4·66 |  | 5052 |  | 7·9 | 7·0 | 8·8 |  | 400 | 355 | 444 |
| *Male deaths at age 35-74 years* | | | | | | | | | | | |  | *10 604* |  | *68·1* | *60·5* | *75·7* |  | *7220* | *6418* | *8022* |
| *Male deaths at age ≥35 years* | | | | | | | | | | | |  | *13 013* |  | *56·7* | *50·4* | *63·0* |  | *7381* | *6561* | *8201* |
| *Male deaths at all ages* | | | | | | | | | | | |  | *15 890* |  | *46·5* | *41·3* | *51·6* |  | *7381* | *6561* | *8201* |
|  |  |  |  |  |  |  |  |  |  |  |  |  |  |  |  |  |  |  |  |  |  |
| **Females** |  |  |  |  |  |  |  |  |  |  |  |  |  |  |  |  |  |  |  |  |  |
| 35-44 years | 0·481 | 0·225 | 0·294 |  | 3·62 | 1·53 | 8·55 |  | 6·92 | 2·98 | 16·04 |  | 1239 |  | 69·7 | 62·0 | 77·5 |  | 864 | 768 | 960 |
| 45-64 years | 0·437 | 0·254 | 0·309 |  | 3·62 | 1·53 | 8·55 |  | 6·92 | 2·98 | 16·04 |  | 4477 |  | 68·8 | 61·2 | 76·5 |  | 3082 | 2739 | 3424 |
| 65-74 years | 0·208 | 0·264 | 0·528 |  | 2·53 | 1·18 | 5·45 |  | 5·51 | 2·36 | 13·15 |  | 2409 |  | 51·6 | 45·8 | 57·3 |  | 1242 | 1104 | 1380 |
| ≥65 years | 0·166 | 0·401 | 0·433 |  | 0·95 | 0·50 | 1·78 |  | 1·97 | 0·83 | 4·66 |  | 3329 |  | 11·1 | 9·9 | 12·4 |  | 370 | 329 | 411 |
| *Female deaths at age 35-74 years* | | | | | | | | | | | |  | *8125* |  | *63·9* | *56·8* | *70·9* |  | *5188* | *4611* | *5764* |
| *Female deaths at age ≥35 years* | | | | | | | | | | | |  | *11 454* |  | *48·5* | *43·1* | *53·9* |  | *5558* | *4941* | *6176* |
| *Female deaths at all ages* | | | | | | | | | | | |  | *13 181* |  | *42·2* | *37·5* | *46·9* |  | *5558* | *4941* | *6176* |
|  |  |  |  |  |  |  |  |  |  |  |  |  |  |  |  |  |  |  |  |  |  |
| **Persons** |  |  |  |  |  |  |  |  |  |  |  |  |  |  |  |  |  |  |  |  |  |
| *Total deaths at age 35-74 years* | | | | | | | | | | | |  | *18 729* |  | *66·2* | *58·9* | *73·6* |  | *12 408* | *11 029* | *13 786* |
| *Total deaths at age ≥35 years* | | | | | | | | | | | |  | *24 467* |  | *52·9* | *47·0* | *58·8* |  | *12 939* | *11 501* | *14 377* |
| *Total deaths at all ages* | | | | | | | | | | | |  | *29 071* |  | *44·5* | *39·6* | *49·5* |  | *12 939* | *11 501* | *14 377* |

RR = Relative Risk. LCI = Lower Confidence Interval. UCI = Upper Confidence Interval.

Estimates are presented for the 35-44 year age group, under the assumption that this group experiences the same RR as the 45-64 year age group.
Current smoking prevalence includes daily and non-daily smokers; the vast majority of current smokers are daily smokers.
a. National estimates of annual total deaths by sex were extracted from ABS Stat. Age-group-specific deaths data are only available for five of the eight States/Territories in Australia (New South Wales, Queensland, South Australia, Western Australia and the Northern Territory) due to data quality limitations. To estimate national age-sex-group deaths, we applied a scale factor (total number of deaths by sex / five State-Territory deaths by sex) to the number of deaths in each age-sex-group in the five State-Territory data.
b. According to assumptions that 90%, 80%, and 100% of excess deaths among smokers are smoking-attributable. We calculated SAF using the prevalence-based method, for the age groups 35-44, 45-54, 55-64, and ≥75 years: SAF(%)=100x[P_p_(RR_p_-1)+P_c_(RR_c_-1)]/[P_p_(RR_p_-1)+P_c_(RR_c_-1)+1]. Here, P_p_ and P_c_ are the prevalence of past and current smoking, respectively, and RR_p_ and RR_c_ are the RR^2^s for mortality among past and current smokers, respectively, compared to never-smokers. The Smoking Attributable Fraction is calculated using sex-combined RR^2^ results for participants aged 45-64 (also applied to the 35-44 year age group), 65-74 and ≥75 years, adjusted for age as the underlying time variable, sex, education and remoteness.

**Table S9. Smoking attributable fraction for the near future, based on 2018-19 smoking prevalence, for adults aged ≥45 years**

|  | **2018-19 smoking status (proportion)** | | |  | **Past smoker: sex-combined mortality RR** | | |  | **Current smoker: sex-combined RR** | | |  |  | **Smoking Attributable Fraction^a^** | | |
| --- | --- | --- | --- | --- | --- | --- | --- | --- | --- | --- | --- | --- | --- | --- | --- | --- |
|  | **Current** | **Past** | **Never** |  | **RR** | **LCI** | **UCI** |  | **RR** | **LCI** | **UCI** |  |  | **Estimate (∝=0·90)** | **Lower bound (∝=0·80)** | **Upper bound (∝=1·00)** |
| **Males** |  |  |  |  |  |  |  |  |  |  |  |  |  |  |  |  |
| 45-64 years | 0·463 | 0·287 | 0·250 |  | 3·62 | 1·53 | 8·55 |  | 6·92 | 2·98 | 16·04 |  |  | 70·0 | 62·2 | 77·8 |
| 65-74 years | 0·249 | 0·574 | 0·176 |  | 2·53 | 1·18 | 5·45 |  | 5·51 | 2·36 | 13·15 |  |  | 60·0 | 53·4 | 66·7 |
| ≥75 years | 0·150 | 0·580 | 0·270 |  | 0·95 | 0·50 | 1·78 |  | 1·97 | 0·83 | 4·66 |  |  | 10·2 | 9·1 | 11·3 |
|  |  |  |  |  |  |  |  |  |  |  |  |  |  |  |  |  |
| **Females** |  |  |  |  |  |  |  |  |  |  |  |  |  |  |  |  |
| 45-64 years | 0·445 | 0·298 | 0·257 |  | 3·62 | 1·53 | 8·55 |  | 6·92 | 2·98 | 16·04 |  |  | 69·6 | 61·9 | 77·3 |
| 65-74 years | 0·215 | 0·458 | 0·327 |  | 2·53 | 1·18 | 5·45 |  | 5·51 | 2·36 | 13·15 |  |  | 56·3 | 50·1 | 62·6 |
| ≥75 years | 0·172 | 0·353 | 0·476 |  | 0·95 | 0·50 | 1·78 |  | 1·97 | 0·83 | 4·66 |  |  | 11·7 | 10·4 | 13·0 |

RR = Relative Risk. LCI = Lower Confidence Interval. UCI = Upper Confidence Interval.

Current smoking prevalence includes daily and non-daily smokers; the vast majority of current smokers are daily smokers.
a**.** According to assumptions that 90%, 80%, and 100% of excess deaths among smokers are smoking-attributable. We calculated SAF using the prevalence-based method: SAF(%)=100x[p_ps_(RR_ps_-1) + p_cs_(RR_cs_-1)] / [p_ps_(RR_ps_-1) + p_cs_(RR_cs_-1) + 1]. Here, p_ps_ and p_cs_ are the prevalence of past and current smoking, respectively, and RR_ps_ and RR_cs_ are the RR^2^s for mortality among past and current smokers, respectively, compared to never-smokers. The Smoking Attributable Fraction is calculated using sex-combined RR^2^ results for participants aged 45-64, 65-74 and ≥75 years, adjusted for age as the underlying time variable, sex, education and remoteness.

**Table S10. Second-hand smoke (SHS) exposure at baseline among Aboriginal participants in the 45 and Up cohort, overall and by smoking status**

|  | **Smoking Status** | | | | | |  | **Total** | |
| --- | --- | --- | --- | --- | --- | --- | --- | --- | --- |
|  | **Current** | | **Past** | | **Never** | |  |  |  |
|  | **%** | **(n)** | **%** | **(n)** | **%** | **(n)** |  | **%** | **(n)** |
| **Overall** | 21·5 | 298 | 36·6 | 509 | 41·9 | 582 |  | 100 | 1389 |
| **SHS exposure at home** |  |  |  |  |  |  |  |  |  |
| No exposure | 33·2 | (99) | 62·8 | (319) | 66·2 | (385) |  | 57·9 | (803) |
| Any exposure | 37·6 | (112) | 12·0 | (61) | 9·3 | (54) |  | 16·4 | (227) |
| Missing | 29·2 | (87) | 25·2 | (128) | 24·6 | (143) |  | 25·8 | (358) |
| **SHS exposure in other places** |  |  |  |  |  |  |  |  |  |
| No exposure | 25·2 | (75) | 43·7 | (222) | 48·5 | (282) |  | 41·7 | (579) |
| Any exposure | 42·3 | (126) | 38·0 | (193) | 33·2 | (193) |  | 36·9 | (512) |
| Missing | 32·6 | (97) | 18·3 | (93) | 18·4 | (107) |  | 21·4 | (297) |
| **Total exposure to SHS** |  |  |  |  |  |  |  |  |  |
| No exposure | 21·1 | (63) | 44·5 | (226) | 48·1 | (280) |  | 41·0 | (569) |
| >0 to <1 hour per day | 21·1 | (63) | 28·5 | (144) | 25·6 | (149) |  | 25·6 | (356) |
| ≥1 hours per day | 38·9 | (116) | 15·4 | (78) | 12·2 | (71) |  | 19·1 | (265) |
| Missing | 18·8 | (56) | 11·8 | (60) | 14·1 | (82) |  | 14·3 | (198) |

SHS = second-hand smoke.

**Table S11. Aboriginal and Torres Strait Islander age-specific mortality rates, national (five States and Territories) and NSW, 2018 and age-specific crude mortality rates and 95%CIs in the 45 and Up cohort**

|  |  | **Age-specific death rate (per 1000)** | | | |
| --- | --- | --- | --- | --- | --- |
|  |  | **Aboriginal and Torres Strait Islander population** | |  | **Aboriginal 45 and Up Study participants**  **(95%CI)** |
| **Age group (years)** |  | **National (five States/Territories)** | **NSW** |  |  |
| 45 - 54 |  | 6·84 | 5·03 |  | 4·90 (3·57-6·73) |
| 55 - 64 |  | 12·50 | 8·95 |  | 9·77 (7·32-13·04) |
| 65 - 74 |  | 25·06 | 18·55 |  | 23·46 (17·27-31·86) |

95%CI = 95% Confidence Interval.

2018 data on Aboriginal and Torres Strait Islander deaths nationally (limited to the five States/Territories with adequate data quality) and in New South Wales.

**Table S12. Smoking attributable fraction during cohort follow up, and smoking-attributable deaths 2009-2018 adjusted for misclassification of deaths**

|  | **Estimated % of deaths misclassified** |  | **Adjusted total number of deaths 2009-2018^a^** |  | **Adjusted smoking-attributable deaths nationally 2009-2018^b^** | | |
| --- | --- | --- | --- | --- | --- | --- | --- |
|  |  |  |  |  | **Estimate (∝=0·90)** | **Lower bound (∝=0·80)** | **Upper bound (∝=1·00)** |
| **Males** |  |  |  |  |  |  |  |
| 45-64 years | 12% |  | 6779 |  | 4781 | 4249 | 5312 |
| 65-74 years | 12% |  | 2958 |  | 1768 | 1571 | 1964 |
| ≥75 years | 19% |  | 2874 |  | 227 | 202 | 253 |
| *Total males ≥45* |  |  | *12 610* |  | *6859* | *6096* | *7621* |
|  |  |  |  |  |  |  |  |
| **Females** |  |  |  |  |  |  |  |
| 45-64 years | 11% |  | 4969 |  | 3421 | 3041 | 3801 |
| 65-74 years | 11% |  | 2674 |  | 1379 | 1226 | 1532 |
| ≥75 years | 18% |  | 3918 |  | 436 | 387 | 484 |
| *Total females ≥45* |  |  | *11 562* |  | *5313* | *4723* | *5904* |
|  |  |  |  |  |  |  |  |
| **Persons** |  |  |  |  |  |  |  |
| *Total persons ≥45* |  |  | *24 172* |  | *12 170* | *10 818* | *13 523* |

Data on misclassification is drawn from *Australian Institute of Health and Welfare (2019). Improving Indigenous identification in mortality estimates. Canberra, AIHW.* These estimates are adjusted according to the extent of misclassification identified in analysis of the Enhanced Mortality Database; this analysis assumes that a similar extent of misclassification has occurred over the relevant period (2009-2018) as compared to the period of their analysis (2001-2015).

a. National estimates of annual total deaths by sex were extracted from ABS Stat. Age-group-specific deaths data are only available for five of the eight States/Territories in Australia (New South Wales, Queensland, South Australia, Western Australia and the Northern Territory) due to data quality limitations. To estimate national age-sex-group deaths, we applied a scale factor (total number of deaths by sex / five State-Territory deaths by sex) to the number of deaths in each age-sex-group in the five State-Territory data.

b. According to assumptions that 90%, 80%, and 100% of excess deaths among smokers are smoking-attributable.

**Figure S1.** **Flow chart showing participants excluded and included in the study sample.**


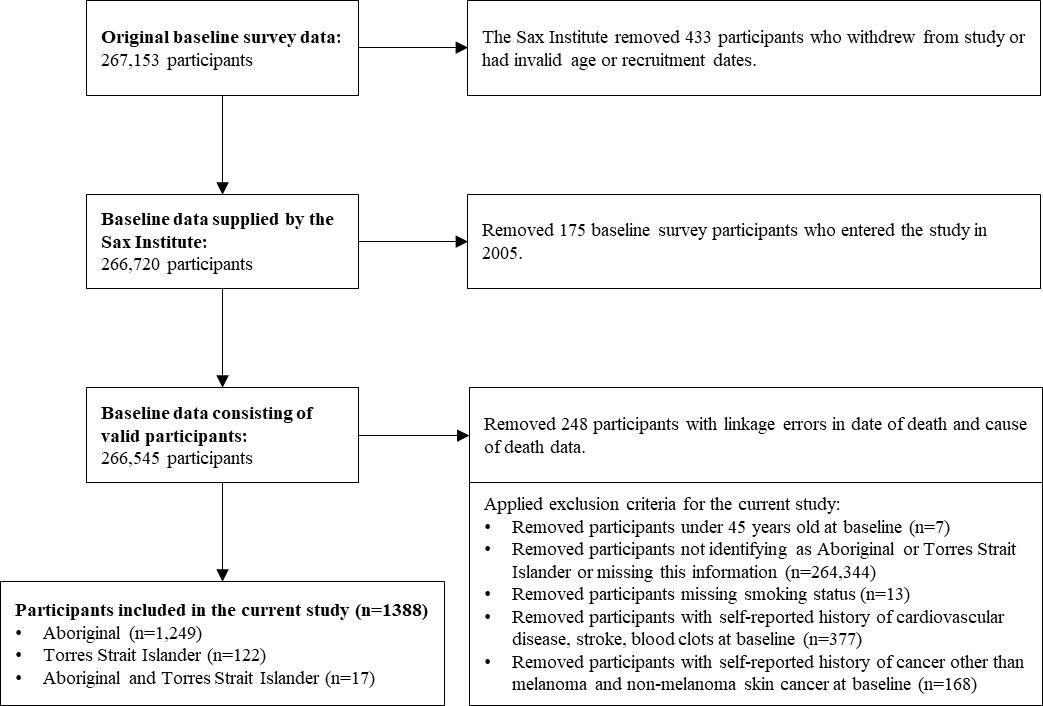

Supplement: dyaa274_Supplementary_Data [file dyaa274_supplementary_data.docx]
